# HSPB1 Facilitates the Formation of Non-Centrosomal Microtubules

**DOI:** 10.1371/journal.pone.0066541

**Published:** 2013-06-24

**Authors:** Leonardo Almeida-Souza, Bob Asselbergh, Vicky De Winter, Sofie Goethals, Vincent Timmerman, Sophie Janssens

**Affiliations:** 1 Department of Molecular Genetics, VIB and University of Antwerp, Antwerpen, Belgium; 2 Neurogenetics Laboratory, Institute Born Bunge, University of Antwerp, Antwerpen, Belgium; 3 GROUP-ID Consortium, Laboratory for Immunoregulation and Mucosal Immunology, University of Ghent, Ghent, Belgium; 4 Department of Molecular Biomedical Research, VIB, Ghent, Belgium; Stanford University School of Medicine, United States of America

## Abstract

The remodeling capacity of microtubules (MT) is essential for their proper function. In mammals, MTs are predominantly formed at the centrosome, but can also originate from non-centrosomal sites, a process that is still poorly understood. We here show that the small heat shock protein HSPB1 plays a role in the control of non-centrosomal MT formation. The HSPB1 expression level regulates the balance between centrosomal and non-centrosomal MTs. The HSPB1 protein can be detected specifically at sites of *de novo* forming non-centrosomal MTs, while it is absent from the centrosomes. In addition, we show that HSPB1 binds preferentially to the lattice of newly formed MTs *in vitro*, suggesting that its function occurs by stabilizing MT seeds. Our findings open new avenues for the understanding of the role of HSPB1 in the development, maintenance and protection of cells with specialized non-centrosomal MT arrays.

## Introduction

The small heat shock protein HSPB1 (also known as HSP27) is a ubiquitously expressed molecular chaperone that besides its canonical folding function is involved in a wide range of cellular processes such as apoptosis, actin cytoskeleton regulation and cell proliferation [1]. Due to its multifunctional character, HSPB1 was shown to play a role in several human disorders including cancer, Alzheimer’s and heart disease [2]. In 2004 we found that mutations in HSPB1 cause a peripheral neuropathy called Charcot-Marie-Tooth disease (CMT) [3].

Previously we discovered that a subset of these CMT causing HSPB1 mutations leads to higher chaperone activity [4] and an increased binding of HSPB1 to tubulin and MTs [5]. This enhanced binding stabilizes the MT network in a similar fashion to what is described for other microtubule associated proteins (MAPs) [6]. Interestingly, we found that wild-type HSPB1 also binds tubulin and MTs, which confirms earlier data [7], [8]. However, the functional relevance of this binding has never been addressed. Here, we show that HSPB1 regulates the architecture of the cellular MT network by facilitating the formation of non-centrosomal MTs.

## Materials and Methods

### Cell Culture, Constructs and Recombinant Protein Production

Stable HeLa and CHO cell lines were produced by lentiviral transduction according to the method described in [9]. Cells with HSPB1 overexpression were produced with constructs containing V5 tagged HSPB1 using the constructs described elsewhere [4]. HeLa cells with HSPB1 knock down were generated by lentiviral transduction of shRNA constructs (Sigma). Stable cell lines were discarded and freshly generated after 15–25 passages to ensure optimal expression and silencing of HSPB1. Most experiments were repeated using stable cell lines generated independently. TUBB3-GFP and EB1-GFP constructs were generated using Gateway recombination (Invitrogen). For recombinant protein production, the ORF from HSPB1 was amplified by PCR and cloned by topoisomerase cloning into the vector pCRT7-NT-TOPO (Invitrogen). Protein expression was induced in BL21 DE3 cells and proteins were purified on a Ni-NTA column followed by a size exclusion purification step in an S200 column. DCX was purchased from Origene.

### Nocodazole Washout Experiments

**Figure 1:**
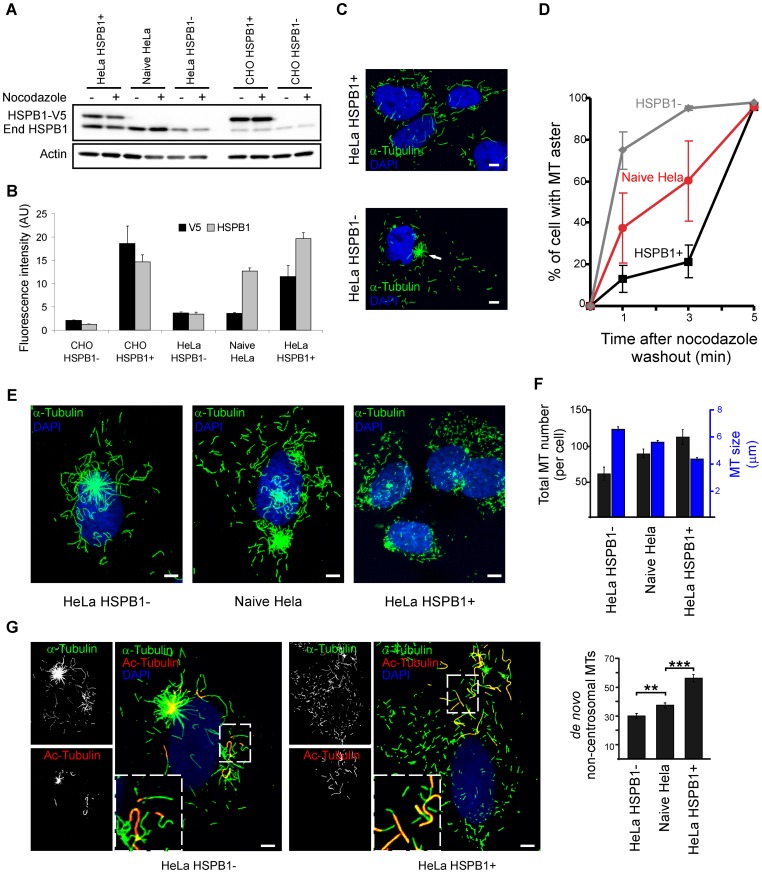
HSPB1 facilitates the formation of non-centrosomal microtubules in HeLa cells. (A) Western blot using a polyclonal anti-HSPB1 antibody showing different HSPB1 expression levels in HeLa and CHO cells before and after nocodazole treatment. These were obtained by using either non-transduced cells (called naive Hela and CHO HSPB1-), cells transduced with HSPB1-V5 (called HeLa HSPB1+ and CHO HSPB1+) or cells transduced with shRNA HSPB1 constructs (called HeLa HSPB1-). End HSPB1 = endogenous HSPB1. (B) Fluorescence quantification of HSPB1 expression using anti-V5 and anti-HSPB1 antibodies in different HeLa and CHO stable cell lines (V5/HSPB1 staining n = 33/56, 63/58, 36/48, 50/41, 32/53 for CHO HSPB1-, CHO HSPB1+, HeLa HSPB1−, Naive HeLa and HeLa HSPB1+, respectively). (C) Representative images from HeLa HSPB1+ and HeLa HSPB1- cells at 1 min after nocodazole washout. The white arrow indicates a MT aster that is present predominantly in HeLa HSPB1- cells. (D) Quantification of the percentage of cells containing a MT aster at different time points after nocodazole treatment (data from 3 independent experiments of at least 100 cells). (E) The MT distribution at 5 min after nocodazole washout showing a different repolymerization pattern between cells with different HSPB1 levels. (F) Quantification of the length and amount of total MTs cells expressing different amounts of HSPB1 at 5 min after nocodazole treatment (n = 24, 20, 20 cells for HSPB1+, Naive Hela and HSPB1-, respectively). (G) Representative images of HeLa HSPB1- and HeLa HSPB1+ stained for α- and acetyl-tubulin. For the quantifications shown in the graph, only MTs not showing acetylated regions were counted (n = 49, 64, 58 cells for HSPB1+, Naive Hela and HSPB1− respectively). In F and G, all averages are different from each other with p>0.05, with the exception of total MT number between HeLa HSPB1+ and Naive HeLa (p = 0.09). Data is presented as average ± SEM. Scale bars = 5 µm.

Cells were seeded in 24-well plates containing coverslips and treated 48 h later with 10 µm nocodazole for 6 h. After treatment, cells were washed once with cold PBS and fresh, pre-warmed media was added and cells were allowed to recover for different periods. After the recovery period, media was removed and unpolymerized tubulin was washed away by detergent extraction (BRB80 containing 0.5% Triton X-100 for 30 s). For colocalization experiments that include HSPB1 staining, we omitted the detergent extraction step because it removes all HSPB1 from the cells. Cells were then fixed with 3% paraformaldehyde (PFA) and stained with the indicated antibodies by standard immunocytochemical procedures. Following primary antibodies were used: Mouse monoclonal anti-α-tubulin (Abcam), Mouse monoclonal anti-α-tubulin conjugated with dyomics-547 (Abcam), Mouse monoclonal anti-HSPB1 (Enzo Life Sciences), Goat polyclonal anti-HSPB1 (Santa Cruz biotechnology), Mouse monoclonal anti-acetylated-tubulin (Santa Cruz biotechnology), Rabbit polyclonal anti-Giantin (Abcam). When α-tubulin and acetylated tubulin were co-stained (as in Fig. 1F), the mouse anti-acetylated-tubulin antibody and corresponding secondary antibody was used prior to staining with the directly conjugated mouse anti-α-tubulin antibody to avoid cross-reactivity.

Confocal image stacks were taken with a Zeiss LSM700 confocal laser scanning microscope equipped with a 63X 1.4 NA objective and 405, 488, 555 and 639 nm diode lasers. Standard excitation and emission settings were used to detect and discriminate the different fluorophores. When different experimental conditions were compared, identical acquisition parameters were used for all conditions.

If indicated, image hyperstacks (xyzt) were deconvolved using the ImageJ Iterative Deconvolve 3D plugin with theoretically calculated point spread functions, created with the Diffraction PSF CD plugin (Copyright (c) 2005, OptiNav, Inc., http://www.optinav.com/Iterative-Deconvolve-3D.htm and http://www.optinav.com/Diffraction-PSF-3D.htm).

For measurement of total MT length and number of MTs in HeLa cells, images were initially processed with the *Tubeness* plugin of the open source image analysis platform ImageJ [10]. This filter recognizes tubular structures and was used to improve the sharpness of MTs and to remove the central part of centrosomes (as they are not recognized as tubular structures), facilitating the counting. We then applied the skeletonize filter to transform MTs in single pixel lines and the number and size of MTs was determined using the ImageJ *3D Object Counter* plugin. The segmentation results were manually verified and a size threshold was determined for each image to remove from the analysis overlapping MTs that were erroneously considered as single MTs.

D*e novo* non-centrosomal formed MTs in HeLa cells were counted manually. Only MTs without any sign of tubulin acetylation were considered a truly *de novo* formed MT. Counting of cells with clear MT asters in HeLa cells was performed by manual scoring. Three independent groups of at least 100 cells were counted per coverslip. The size of MT asters in CHO cells were determined by manually selecting the MT asters and measuring its size in ImageJ.

### Live Cell Imaging

CHO cells were cultivated in 35 mm glass-bottom dishes and transfected with a EB1-GFP construct [5] using lipofectamine LTX (Invitrogen). Cells were imaged 72 h after transfection using using an Ultra*VIEW* VoX microlens-enhanced dual spinning disk (Yokogawa CSU-X1) system (PerkinElmer), installed on a Nikon Ti inverted microscope equipped with a C9100-50 EMCCD camera (Hamamatsu) and controlled by Volocity software (PerkinElmer). For EGFP discrimination, 488 nm diode laser excitation and standard fluorescence filters were used in combination with a 60X 1.4 NA plan apochromatic objective. The Nikon Perfect Focus System (“PFS”) was used to automatically maintain the microscope focus. During imaging, cells were maintained at 37°C and 5% CO_2_.

For live observation of MT repolymerization, cells were treated with nocodazole for 6 h, placed in the microscope and washed three times with 5 ml of media while imaging. Multi-plane image z-stacks comprising the complete cell volume were acquired every 5 sec, with a total exposure time of the stack of less than 2 sec. After acquisition, image stacks were exported as 16bit Tiff files and further processed in ImageJ. To visualise the dynamics of the entire 3D volumes in a single plane, maximum intensity projections were created for every time point, which were then combined into a single time sequence. As the duration of washings varied between videos, we decided to set as time zero the moment where the first EB1-GFP fluorescence bursts occurred. Non-centrosomal EB1 comets were tracked using the ImageJ *Manual Tracking* plugin. Only comets with unambiguous non-centrosomal origin that could be clearly followed for more than three frames were tracked.

For observation of EB1-GFP comets in steady state, image acquisition and image processing was performed as described above, except for higher camera exposure times and smaller time intervals: multi-plane images of cells were acquired for 2 min at a 3 sec/frame. To distinguish between centrosomal and non-centrosomal MTs, we imaged only cells in which the centrosome was localized between the nucleus and the glass substrate and clearly distinguishable from the perinuclear Golgi area. EB1 comets were tracked using the ImageJ *Manual Tracking* plugin. To ensure that only bona fide centrosomal and non-centrosomal EB1 comets were shown, we ignored comets with ambiguous/unknown origin or that could not be individualized from other tracks.

### MT Distribution Measurement

To evaluate the relative distribution of tubulin over the center and the cell periphery, a customized image analysis procedure was generated in ImageJ to measure pixel intensities near the cell center and near the cell periphery. Two regions of interest were manually selected for each cell: i) a central point, inside the region with highest tubulin concentration and located centrally in the cell, was identified as a point selection; and ii) the borders of the cell were outlined with a polygon selection. Next, the ImageJ macro uses the coordinates of these regions of interest to generate a number of straight line profiles, starting at the central point and ending at the cell border. The lines, which will differ in length, are created for different angles with a fixed angle interval and together comprise the full 360° rotation. In the results that are presented, 120 lines are drawn with an angle interval of 3°. For each of the line profiles two parameters are measured: i) the mean pixel intensity in the inner part of the line (closest to the central part of the cell; in the presented data defined as the inner 10% of the line length), and the mean pixel intensity of the outer part of the line (near the cell periphery; defined in the presented data as the outer 40% of the line length). The ratio (R) between the inner (I_i_) and the outer (I_o_) mean intensities is then calculated for each line and the microtubule distribution index (MDI) is represented by the average of all R’s for each cell. The MDI is a parameter that correlates with the tubulin distribution in the cell: a low ratio represents a predominantly centralized MT network, whereas a higher ratio corresponds to a more spread out distribution of the MT network. Variations in the measurement procedure were tested, such as using different angle intervals, taking larger or smaller inner and outer line lengths or measuring maximum instead of mean pixel intensities. These changes in settings resulted in similar outcomes for the output ratios, hence, these were chosen somewhat arbitrarily for the presented data.

The measurements were performed on maximum intensity projections of confocal z-stacks, acquired in a Zeiss LSM700 confocal microscope equipped with a 63X 1.4 NA objective with identical acquisition parameters for both genotypes. In total, the measurements were performed on over 200 cells for each genotype, originating from three independent tubulin stainings.

### Microtubule Dynamics Measurements

HeLa cells with different HSPB1 levels were cultivated in 35 mm glass-bottom dishes and transfected with the TUBB3-GFP constructs using Lipofectamine LTX (Invitrogen). Cells were imaged 72 h after transfection for 2 min at 3 sec per frame for TUBB3-GFP-transfected cells on a Zeiss Axiovert 200 microscope with a microlens-enhanced dual spinning disc confocal system (UltraVIEW ERS; PerkinElmer), equipped with a 63X 1.4NA objective. During imaging, cells were maintained at 37°C and 5% CO_2_. Individual TUBB3-GFP MTs were tracked manually using the *Manual Tracking* plugin of ImageJ and dynamics parameters were extracted from the data by a custom-made Perl script. The MTs were considered static if they moved less than 0.3 µm per frame.

### MT Cosedimentation Assay

The binding of HSPB1 to MTs was assessed by polymerizing 37.5 µM pure tubulin (Cytoskeleton Inc) in the presence of 10 µM HSPB1 or 10 µM GFP in BRB80 (80 mM PIPES, 1 mM MgCl_2_, 1 mM EDTA), supplemented with 1 mM GTP for 30 min. After polymerization, reactions were centrifuged at 100.000 g for 30 min at 35°C. Supernatant was then transferred to another tube, the pellet was washed with BRB80 buffer and resuspended in the same volume as the supernatant.

### In vitro Microtubule Polymerization Assays

Microtubule assembly was monitored by DAPI fluorescence according to the protocol described elsewhere [11]. Polymerization was performed using MAP-rich tubulin (Cytoskeleton Inc - Cat. # ML113) in BRB80 buffer and 1 mM GTP and 10 µM DAPI (final concentrations) and recombinant HSPB1 at 5 µM when stated. The fluorescence of MT-bound DAPI was monitored with a BioTek plate reader each 30 sec with the temperature set to 37°C. The excitation and emission wavelengths were set at 360 and 450 nm, respectively.

### In vitro MT Nucleation Assay

A solution in BRB80 (80 mM PIPES, 1 mM MgCl_2_, 1 mM EDTA) of 8 µm tubulin at a 1∶10 ratio of TRITC-rhodamine tubulin:unlabeled tubulin (MAP-rich tubulin - Cytoskeleton Inc) supplemented with 1 mM GTP with different concentrations of HSPB1 and DCX were incubated at 37°C for 5 min, fixed for 3 min with 1% glutaraldehyde in BRB80, diluted 100x in BRB80 and spotted onto coverslips. Images (1024×1024 pixels, 0.1 µm/pixel) were acquired with a Zeiss LSM700 confocal microscope equipped with a 63X 1.4 NA objective. Number and size of MTs were measured by using the ImageJ *Analyze Particles* function after intensity thresholding with a fixed minimum. At least 25 random image fields on the coverslip were used.

### Immuno-EM

10 µm tubulin (MAP-rich tubulin) together with 10 µm HSPB1 or EGFP were polymerized for 3 or 30 min in BRB80/GTP as described above and reactions were stopped by adding glutaraldehyde at a 1% final concentration. The 3 min time point was chosen for containing many short MTs that would resemble a recently nucleated MT, while the 30 min time point contain predominantly long MTs resembling mature MTs. Five µl of the reaction was spotted on formvar carbon coated 200 Mesh EM grids (Electron Microscopy Sciences). Grids were then incubated for 10 min with anti-HSPB1 (Stressgen) or anti-GFP (Clontech) antibodies, washed with 20 µl of BRB80 and incubated for an additional 10 min with goat anti-mouse IgG labeled with 10 nm gold (Aurion). Next, grids were stained with uranyl acetate and visualized in a FEI tecnai G2 Spirit BioTWIN transmission electron microscope (FEI Co) at a 26.500×magnification. The MT length was measured using ImageJ and quantification of HSPB1 binding density to MTs was done by visual counting. For the calculation of the correlation between MT-bound HSPB1 density and MT length, a series of overlapping EM images (4×4 at 26.500×magnification) of MTs at the 3 min polymerization condition were stitched together using the ImageJ plugin *MosaicJ* [12]. MT length and MT-bound HSPB1 density was calculated as above, using only MTs fully contained within the stitched image. Correlation was calculated using Spearman’s correlation coefficient.

### Statistical Analyses

Statistical significance for the different measurements was tested using a two-tailed t-test.

## Results

### HSPB1 Favors the Formation of Non-centrosomal Microtubules

While studying the role of CMT-causing HSPB1 mutants in MT biology [5] we observed that HeLa cells overexpressing HSPB1 (either mutant or wild-type forms) display a less prominent MT aster when compared to their naive, untransfected counterparts during repolymerization of the MT network. To investigate this observation in more detail we repeated these experiments using stable HeLa cells expressing different levels of HSPB1 by using either overexpression or knockdown of HSPB1 (hereafter called HeLa HSPB1+ and HeLa HSPB1- cells, respectively) (Fig. 1A and Fig. 1B). The MT network was depolymerized by nocodazole treatment for 6 h and allowed to repolymerize for short periods of time prior to fixation and analysis. In most mammalian cells, MTs repolymerize by eradiating from the centrosome in a distinct aster-like structure. However, at 1 min after nocodazole washout, only a small fraction of the HeLa HSPB1+ cells (∼10%) displayed a distinct centrosomal MT aster while most HeLa HSPB1− cells (∼80%) showed a distinct centrosomal MT aster (Fig. 1C). Naive HeLa cells, which only express endogenous HSPB1, exhibited an intermediate phenotype with distinct MT asters in about 40% of cells. At later stages of repolymerization, the number of cells presenting clear asters increased steadily for all conditions until all cells showed clear asters at 5 min after nocodazole washout (Fig. 1D).

In addition to the effect on the MT aster, we observed that HeLa HSPB1+ cells displayed a large number of small isolated non-centrosomal MTs spread in all regions of the cytoplasm at 5 min post-nocodazole washout, while HeLa HSPB1- cells presented fewer and longer MTs eradiating mostly from the centrosomal area. Again, naive HeLa cells showed an intermediate phenotype (Fig. 1E). Quantification of length and number of total MTs showed that the number of MTs was positively related to the expression level of HSPB1, while MT length presented an inverse trend (Fig. 1F).

During these experiments we observed that in many cells, even after 6 h of nocodazole treatment, a few short MTs remain intact, which could consequently also serve as templates for repolymerization after nocodazole washout. To correctly identify and quantify only *de novo* formed non-centrosomal MTs, we included an additional co-staining for acetyl-tubulin to discriminate MTs that were unaffected by the depolymerization treatment. Immediately after nocodazole washout, all resident MTs were completely acetylated and did not differ in number, length nor distribution between HeLa HSPB1+ and HeLa HSPB1− cells (Fig. S1). During repolymerization, the number of *de novo* formed non-centrosomal MTs, i.e. MTs not harboring an acetylated tail, correlated to HSPB1 levels (Fig. 1G). Interestingly, cells expressing the CMT causing HSPB1 mutant S135F, which is hyperactive [4] and stabilizes mature MTs [5] displayed the same amount of *de novo* non-centrosomal MTs and showed a similar, less prominent aster formation as cells expressing wild-type HSPB1 (HSPB1+) (Fig. S2). These results confirm that HSPB1 does not influence the availability of nocodazole-resistant MT that serve as seeds for polymerization, but increases the number of newly nucleated non-centrosomal MTs. Furthermore, this function of HSPB1 is not affected in hyperactive CMT causing mutants.

**Figure 2:**
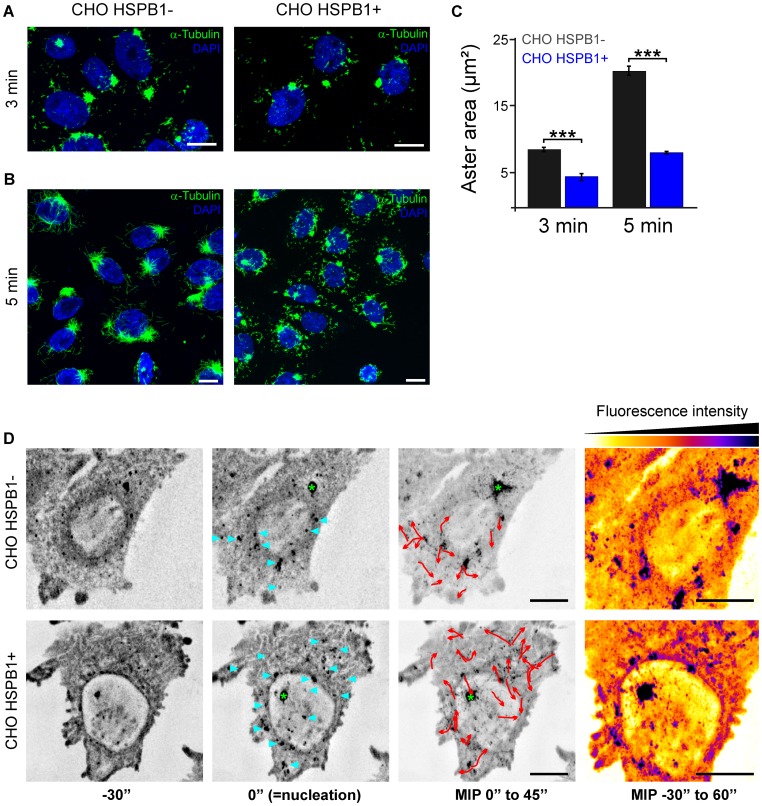
HSPB1 facilitates the formation of non-centrosomal microtubules in CHO cells. Representative images from CHO HSPB1- and CHO HSPB1+ cells at (A) 3 min and (B) 5 min after nocodazole washout. Scale bar = 10 µm. For more examples, see Figure S3. (C) Quantification of the aster area of CHO HSPB1+ and CHO HSPB1- cells at 3 and 5 min after nocodazole washout (n = 90/83 and 106/106 for CHO HSPB1−/CHO HSPB1+ at 3 and 5 min respectively). Data is presented as average ± SEM. (D) Time lapse microscopy of EB1-GFP during nocodazole washout and MT polymerization in CHO HSPB1− and CHO HSPB1+ cells (see Movie S1). Image stacks comprising the complete cell volume were continuously acquired every 5 sec before, during and after nocodazole removal by medium exchange. Maximum intensity projections of all stacks were made and selected time points are shown with inverted grey scale. The appearance of the first EB1 fluorescence burst was set as time zero, which occurred within 1 min after the initiation of the washout procedure (middle images). Before the start of nucleation (left image), only static, residual EB1-GFP spots are visible. The images at the third column are maximum intensity projections (MIP) over time of 9 time points (45 sec) after nucleation. Nucleation sites are marked by blue arrowheads. 3D image deconvolution was applied on the image stacks. Red arrows show the EB1-GFP non-centrosomal comets tracked from nucleation sites. Green asterisk marks the centrosome. On the right side, MIP of the whole time sequence color-coded for intensity. Scale bar = 5 µm.

To confirm these results we performed the same assays employing a different cell line (CHO). In contrast to HeLa cells, CHO cells express low endogenous levels of HSPB1 [13], [14]. Therefore, naive CHO cells (hereafter called CHO HSPB1−) have a comparable HSPB1 level to the HeLa cell with HSPB1 knockdown (HeLa HSPB1−). In addition, we generated a stable CHO cell line overexpressing HSPB1-V5 (Fig. 1A and Fig. 1B) (hereafter called CHO HSPB1+). In line with the results shown for HeLa, CHO HSPB1+ cells displayed a less prominent aster (Fig. 2 A–C) accompanied by a larger number of non-centrosomal MTs (Fig. 2B and Fig. S3) when compared to the CHO HSPB1- cells. On the other hand, despite the difference in aster area, all CHO cell lines displayed a clear aster at all the time points tested (data not shown). This difference with the results in HeLa cells can be explained by the observation that the asters from CHO cells do not depolymerize completely, even after 6 hours of nocodazole treatment (Fig. S4).

As an additional confirmation of these results, we decided to look at the MT repolymerization pattern in these cells in real time. To this end, we transfected cells with the MT +TIP protein EB1-GFP, treated them with nocodazole and imaged MT repolymerization during and after nocodazole washout by time-lapse microscopy. Non-centrosomal MT formation sites could be identified by the formation of fluorescence bursts followed by the formation of a typical EB1-GFP comet with direction and origin clearly distinct from those coming from the centrosome [15]. In line with our previous results, cells with high HSPB1 levels displayed multiple MT formation sites after nocodazole washout while cells with low HSPB1 levels presented predominantly centrosomal MTs (Fig. 2D and Movie S1). The difference in the formation of centrosomal MTs was also clear with CHO HSPB1+ cells showing less centrosomal originated EB1 comets compared to CHO HSPB1− cells.

To check if these results are only due to the differences in HSPB1 levels and not secondary effects, we measured protein levels and the cellular distribution of tubulin, actin and γ-tubulin before and after nocodazole. As shown in Fig. 1A, S5 and S6 the level and the cellular distribution of these proteins did not change between cell lines before or after nocodazole treatment.

### HSPB1 Accumulates at Non-centrosomal MT Formation Sites but not at the Centrosome

**Figure 3:**
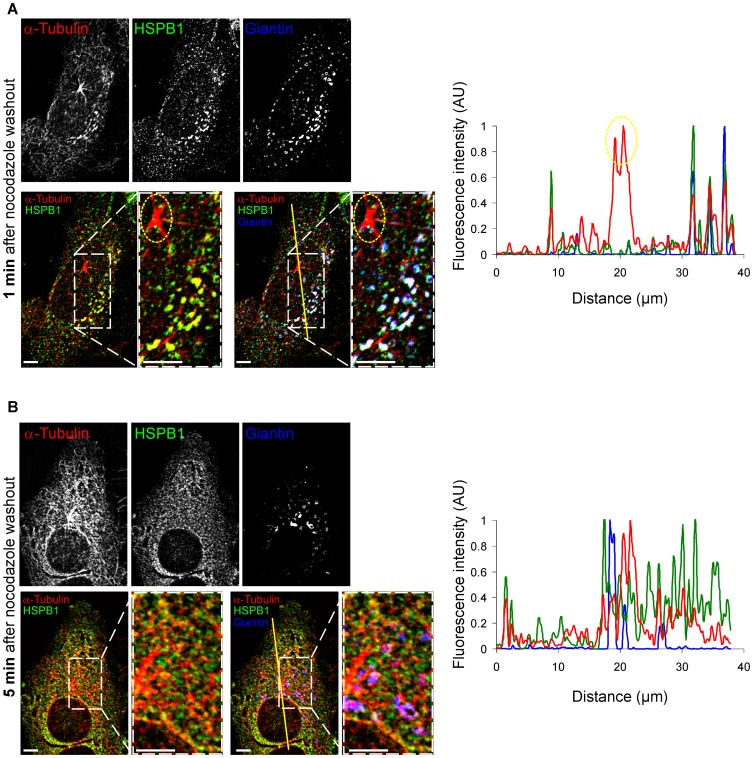
HSPB1 colocalizes to non-centrosomal formation sites only at early stages of repolymerization. Naive HeLa cells stained for HSPB1, α-tubulin and the Golgi apparatus marker Giantin at (A) 1 min and (B) 5 min after nocodazole washout. Graphs represent line intensity plots for the lines drawn in the corresponding images. Note that HSPB1 does not colocalize with the centrosome (circled in yellow). 3D image deconvolution was applied on the image stacks in (A–B). Scale bar = 5 µm.

To further understand the role of HSPB1 in the formation of non-centrosomal MTs and in the growth delay of the MT aster, we investigated the cellular localization of HSPB1 during MT repolymerization after nocodazole treatment in naive HeLa. Consistent with the role of HSPB1 in non-centrosomal MT formation, at 1 min after nocodazole washout, we detected HSPB1 accumulation at perinuclear regions containing high tubulin concentration (Fig. 3A). Interestingly, these tubulin and HSPB1 accumulations resembled in size and localization to the EB1-GFP fluorescence bursts we observed during the live cell imaging (Fig. 2D). Furthermore, these regions also colocalized with the Golgi apparatus, which is a well-known site for non-centrosomal MT formation [16], [17]. Strikingly, while HSPB1 appeared to be present at non-centrosomal sites of MT nucleation, we could not detect any accumulation of HSPB1 at the centrosomes (Fig. 3A), suggesting an indirect influence of HSPB1 on the delay in growth of the MT aster.

Importantly, at 5 min after nocodazole washout, when the MT network is in an advanced state of recovery and the MT formation at the Golgi become less frequent, the accumulation of HSPB1 to the Golgi was less prominent (Fig. 3B).

### HSPB1 Levels Influence Non-centrosomal MT Formation and the MT Network Architecture in Steady State Cells

**Figure 4:**
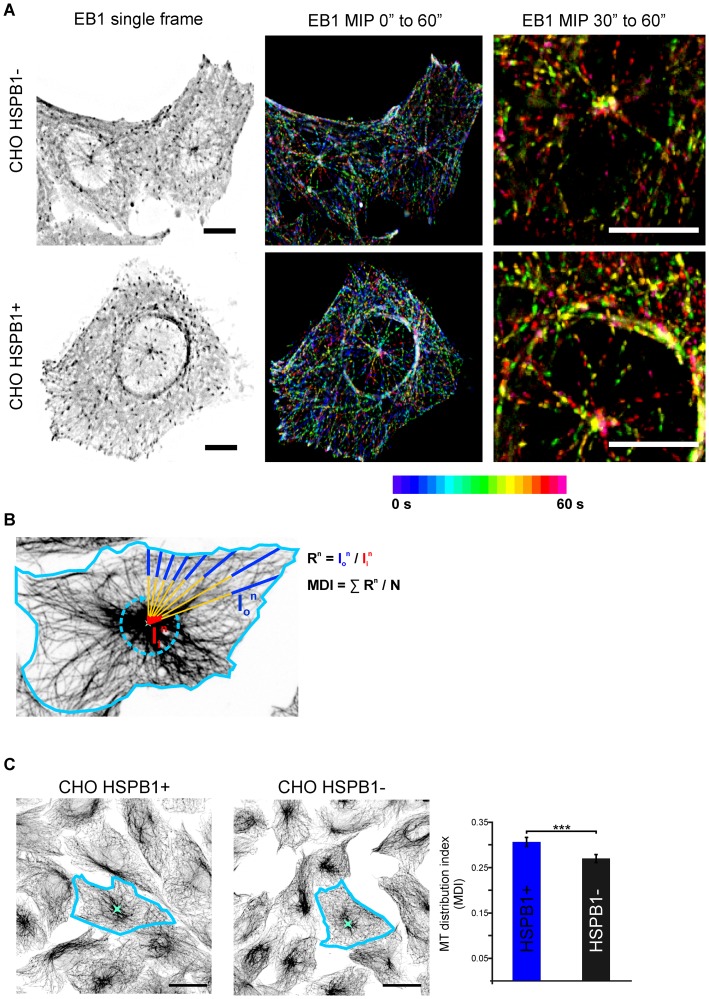
HSPB1 changes the architecture of the MT network in steady state cells. (A) Live cell imaging of CHO cells expressing EB1-GFP (Movie S2). Maximum intensity projections color-coded for time of 20 (center) and 10 (right) frames. (For more examples, see Fig. S7 and Movie S3). Scale bar = 10 µm. (B) Graphical representation of the microtubule distribution index (MDI) method. After defining the cell boundaries and a central intense point in the cell, a series of lines are drawn from the central point to the periphery. Mean intensities from the inner (I_i_) and outer (I_o_) regions of each drawn line were measured. The MDI for each cell is defined as the average I_o_/I_i_ ratio (R) for each line. (C) CHO cells with different HSPB1 levels were stained with α-tubulin and their MDI was calculated (n = >200 cells from three independent stainings). Scale bar = 20 µm.

So far, we have shown that high HSPB1 levels are able to induce the formation of non-centrosomal MTs in cells upon nocodazole treatment. To probe the physiological relevance of these findings we decided to investigate whether HSPB1 also influences MT formation in steady state cells by looking at MT polymerization with EB1-GFP. Because MT rescue events are extremely rare in the center of the cell [18], EB1 tracks originating from central cellular areas can be considered novel nucleation events [17]. Therefore, novel non-centrosomal MT formation events can be identified as EB1 tracks originated from central cellular areas distinct from the regions containing the centrosome. EB1 tracks from cells with high HSPB1 levels showed a clear non-centrosomal pattern, while cells with low HSPB1 levels showed mostly centrosomal-originated tracks, suggesting that HSPB1 levels induce non-centrosomal formation also in steady state (Fig. 4A, Fig. S7, Movies S2 and S3).

Since HSPB1 levels are able to affect the balance between centrosomal and non-centrosomal MT formation, we hypothesized that steady-state cells with high HSPB1 levels would present a more even distribution of MTs throughout the cell, while cells with low HSPB1 levels would present a more concentrated MT distribution due to the predominance of centrosomal MTs. To assess this, we developed an image analysis procedure that measures the distribution of MTs in a cell by calculating the ratio between fluorescence intensities near the centrosome and near the cell periphery (Fig. 4B, see methods for more details). In accordance with our previous results, cells with high levels of HSPB1 displayed a more evenly spread MT network, when compared to cells expressing low HSPB1 levels, which contained a higher proportion of MTs originated from centrosomal regions (Fig. 4C).

Next we also tested whether HSPB1 levels can affect other aspects of MT biology in steady state cells. To this end, we measured MT dynamics parameters using live cell imaging on cells expressing TUBB3-GFP as described previously [5]. As shown in Table S1, the level of HSPB1 had no influence on any of the MT dynamics parameters measured (percentage of time at pause, shrinking or growing states; speed of growth and shrinking; and catastrophe and rescue frequencies). These results contrast our earlier data that show effects of neuropathy causing HSPB1 mutations on MT dynamics [5] and shows that the function of wild-type HSPB1 seems restricted to newly formed MTs.

### HSPB1 Binds Preferentially to the Lattice of Newly Polymerized MTs

**Figure 5:**
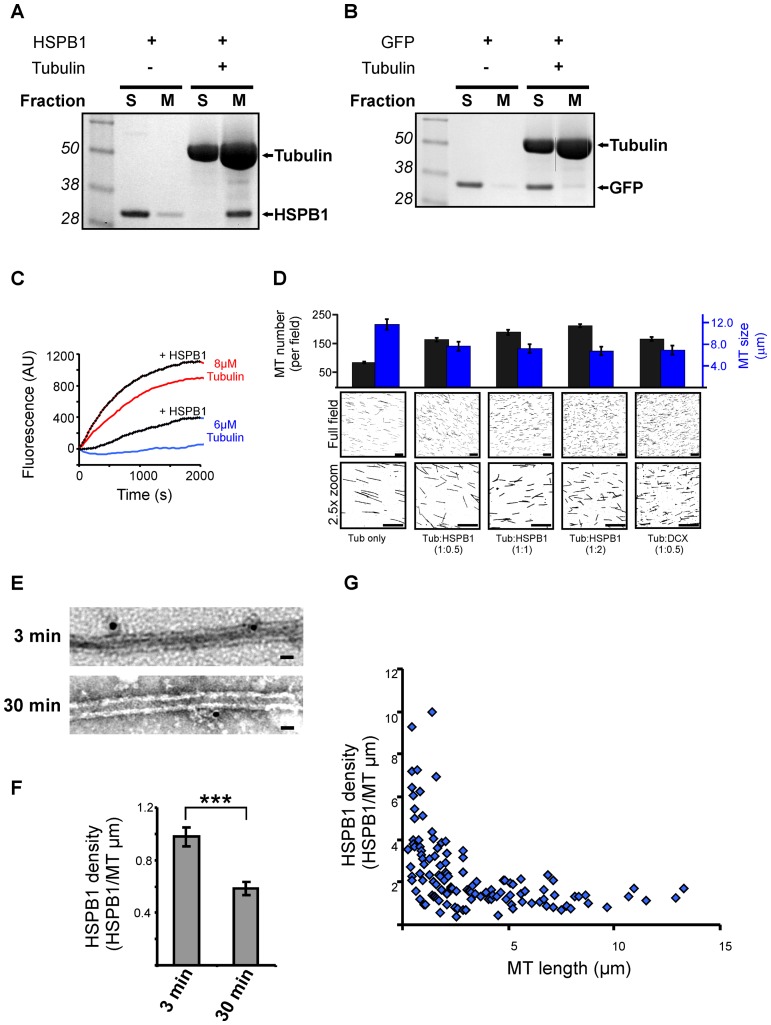
HSPB1 facilitates the formation of MTs in vitro and binds to MTs at early stages of their polymerization. (A–B) Coomassie staining of MT cosedimentation assays. Pure tubulin was polymerized in the presence of (A) HSPB1 or (B) GFP for 30 min and fractionated into MT and soluble proteins fraction. Control reactions without tubulin were also used. S = soluble fraction, M = Microtubule fraction. (C) MTs were polymerized from 8 µM or 6 µM MAP-rich tubulin with or without 5 µM HSPB1. MT assembly was monitored by DAPI fluorescence. (D) MT *in vitro* nucleation assay. 8 µM of a 1∶10 mixture of rhodamine-labeled tubulin and MAP-rich tubulin was polymerized alone or in the presence of different concentrations of recombinant HSPB1 and doublecortin (DCX) for 5 min and spotted onto coverslips. Images from 25 random fields were used to assess number and size of microtubules. All measurements are statistically significant (p<0.01) from each other with the exception of tub:HSPB1(1∶2) and tub:DCX for MT size; and tub:HSPB1(1∶0.5) and tub:DCX for MT number. Scale bar = 20 µm. (E) MTs were polymerized from MAP-rich tubulin in the presence of HSPB1 for 3 and 30 min and visualized by TEM after immunogold staining using an anti-HSPB1 antibody. Scale bar = 50 nm. (E) MTs were polymerized from MAP-rich tubulin in the presence of HSPB1 for 3 and 30 min and visualized by TEM after immunogold staining using an anti-HSPB1 antibody. Scale bar = 50 nm. (F) Quantification of the density of MT-bound gold particles (HSPB1) (n = 105 MTs for 3 min and 110 MTs for 30 min). (G) Scatter plot showing the relationship between MT-bound HSPB1 density and MT length (n = 128 MTs) on MTs polymerized for 3 min. These two parameters showed significant negative correlation (Spearman correlation coefficient = −0.614, p<0.01). Data are presented as average ± SEM. ***p<0.001.

To better understand the role of HSPB1 in MT formation we decided to further study this function *in vitro*. First we checked if the binding of HSPB1 to MTs is direct or mediated by other MAPs using pure tubulin in an *in vitro* MT cosedimentation assay. Tubulin was polymerized in the presence of HSPB1 or the negative control GFP and separated by centrifugation in a MT and a soluble protein fraction. Reactions without tubulin were also used as controls. Confirming that HSPB1 binds directly to MTs, HSPB1 sedimented predominantly in the reaction with MTs (Fig. 5A) while the negative control GFP remained in the soluble fraction for all conditions (Fig. 5B). Next, we checked whether HSPB1 is also able to influence the formation of MTs *in vitro*. MT polymerization was monitored over time using low tubulin concentrations, which disfavors spontaneous nucleation. At a concentration of 8 µM of tubulin, the addition of HSPB1 resulted in significantly more polymerization compared to the condition with tubulin alone. At 6 µM of tubulin, polymerization could only be measured in the reaction containing HSPB1, and was completely absent when HSPB1 was not present (Fig. 5C). To further confirm this finding, we performed an *in vitro* nucleation assay in which a low concentration of tubulin was polymerized to MTs for 5 min in the presence of different amounts of HSPB1 and then spotted on a coverslip to assess MT size, amount and overall structure. MTs from all conditions were formed in a similar dispersed pattern, without the formation of bundles or any aster-like structures. Similarly to what we observed in cells, the presence of HSPB1 resulted in a dose-dependent increase in number and reduction in size of MTs (Fig. 5D). For comparison, we also performed these reactions in the presence of doublecortin (DCX), which was shown before to stabilize and nucleate MTs by lateral binding [19], [20].

We then examined the binding of HSPB1 to MTs by immuno-electron microscopy. To mimic newly formed and mature MTs we polymerized tubulin for a short (3 min) and a long (30 min) period of time before fixation and HSPB1 immunogold staining. As shown in Figure 5E, HSPB1 binds laterally to MTs, similar to what was described for other MAPs [19], [21]. The negative control, using the same conditions and recombinant GFP and an anti-GFP antibody instead, showed little binding to MTs (Fig. S8). In accordance with its exclusive role in newly formed MTs, the density of MT-bound HSPB1 was higher in the condition containing MTs at their initial stage of polymerization (3 min) (Fig. 5E and Fig. 5F). To confirm that the higher binding density of HSPB1 at 3 min does not result from a lower HSPB1:MT ratio in the 30 min reaction, which has more MTs due to extended polymerization time, we plotted the density of MT-bound HSPB1 over the size of MTs within the same reaction (3 min). Confirming our previous result, we found a strong correlation between the density of MT-bound HSPB1 and the length of MTs, with shorter MTs containing a significantly higher density of bound HSPB1 when compared to long mature MTs (Fig. 5G). Taken together, these results show that HSPB1 interacts directly with MTs by lateral binding to the MT lattice, especially in the first stages of MT formation.

## Discussion

Microtubules are cytoskeletal structures characterized by a dynamic behavior consisting of cycles of nucleation, growing and shrinking. The precise spatial and temporal regulation of these cycles is essential for the numerous cellular functions in which MTs are involved. A large number of proteins are capable of regulating this behavior [22]–[24]. Here we add the small heat shock protein HSPB1 to the list of factors regulating the complex biology of MTs. Our results show that HSPB1 binds laterally to newly formed non-centrosomal MTs and facilitates their formation. This increase in non-centrosomal MT formation is accompanied by a reduction in the growth of centrosomal MTs and as such determines the overall architecture of the MT network in steady state cells.

How does HSPB1 assist the formation of MTs? Microtubule nucleation is a complex process in which γ-tubulin, assisted by a large number of accessory proteins, forms a template to which additional tubulin dimers can be sequentially added [25]. These initial polymerized tubulin dimers form an unstable MT seed that must be stable enough to successfully become a mature MT [25]. Our results show that HSPB1 binds preferentially to the lattice of small MTs and, in cells, it concentrates at regions of non-centrosomal nucleation such as the Golgi apparatus [16], [17]. We hypothesize that HSPB1 may be able to either locally increase the concentration of active tubulin necessary for nucleation or recognize and bind unstable lateral protofilament interactions of newly-formed MTs. In either case, HSPB1 would shift the equilibrium towards further polymerization, as opposed to depolymerization, and as such would increase the probability of successful *de novo* MT formation. The fact that HSPB1 binds to MT *in vitro* but cannot be detected after cytosolic extraction suggests that this interaction is weak or that the interaction *in vivo* is very transiently. In addition, HSPB1 could also cooperate with chaperonins and tubulin folding cofactors (TBCs) [26] by helping to activate or stabilize tubulin dimers or other nucleation factors in newly-formed MTs at non-centrosomal sites.

The Golgi apparatus is the most studied non-centrosomal nucleation site in mammalian cells [16], [17], [27]–[29], however other cellular sites were also described to serve as MT nucleation sites such as the nuclear envelope [30] or the plasma membrane [31], [32]. One of the commonalities between all these non-centrosomal nucleation sites is the requirement of γ-tubulin for their function [17], [30], [31]. The wide distribution of MTs formed after nocodazole washout that we observed suggests that not all HSPB1-induced non-centrosomal MTs nucleate from Golgi vesicles, which is confirmed by the fact that only about half of non-centrosomal MTs formed after nocodazole washout were bound to Giantin (data not shown). Still, the phenotype we see in our cells with low HSPB1 levels is remarkably similar to the phenotype of cells depleted with other proteins involved in Golgi-bound MT formation such as CLASP [17], AKAP450 and GM130 [28]. An important question for the future is trying to understand if (and how) these proteins cooperate spatio-temporally to form Golgi-derived MTs.

Besides the HSPB1 function in promoting non-centrosomal MT formation, we also observed that cells with high HSPB1 levels displayed a delayed appearance (in HeLa cells) and reduced growth (in HeLa and CHO cells) of centrosomal MTs. We could not detect any accumulation of HSPB1 at centrosomes, pointing to an indirect negative effect of this protein on MT growth at the centrosome. One plausible explanation is that the large number of non-centrosomal formation sites outcompetes the centrosome for free tubulin, which delays its growth. Alternatively, HSPB1 could also be modulating the levels of other proteins responsible for regulating the growth of centrosomal MTs. The role of HSPB1 controlling the level of other proteins is extensively described in literature [33]–[36].

The role of HSPB1 as a regulator of the actin cytoskeleton has been studied for many years. Initially, HSPB1 was proposed to cap actin filaments and inhibit their polymerization [37]–[39]. However, further work showed that HSPB1 is able to bind to short actin filaments generated after cellular stress in order to avoid their aggregation [40], [41] and to participate in actin filament assembly by shuttling actin monomers in a phospho-dependent manner [42]. In addition, CMT causing mutations in HSPB1 were shown to interfere with the assembly of the neurofilament network [3], suggesting that wild-type HSPB1 may also have a role in the biology of this cytoskeletal structure. Taking into account the results presented in this manuscript, HSPB1 emerges as an important player in the regulation of the cytoskeleton as a whole. Moreover, HSPB1 is present in complexes containing other small heat shock proteins (sHSPs) [43], [44], some of which are also capable to regulate cytoskeletal structures [45]. Understanding how HSPB1, and its sHSPs partners, are regulated and how they cooperate to control all these aspects of cytoskeletal biology will be a great challenge for future research.

We previously showed that peripheral neuropathy causing HSPB1 mutants are hyperactive and bind more strongly to MTs causing their stabilization [5]. In contrast, the hyperactivation of these mutants does not increase their MT formation properties (Fig. S2). Therefore we hypothesize that the MT stabilization property of these mutants is the result of a misregulation of the MT formation activity of wild-type HSPB1. Due to their increased affinity for MTs, we believe that hyperactive mutants remain bound to mature MTs rather than being released after their maturation. This shift in equilibrium between binding and release turns the hyperactive mutants into MAP-like molecules. A graphical representation of this model is presented in Fig. S9.

Our results show that steady state cells with high HSPB1 levels display a more non-centrosomal MT network. In contrast to plants, non-centrosomal MT formation is relatively uncommon in interphasic mammalian cells, but does occur in highly specialized cells, such as neurons, myocytes and epithelial cells [46] Interestingly, these cells constitutively express high levels of HSPB1 [47]–[49] and the knockdown of this protein was shown to be detrimental for their differentiation [49]–[51]. Therefore, it is tempting to speculate that *in vivo* the high levels of HSPB1 may play a role in the formation and maintenance of non-centrosomal MT arrays in these cells. Furthermore, HSPB1 is a known protective factor in heart ischemia [52] and in neurodegeneration [53]. Since the maintenance of the MT network is pivotal in the ability of these cells to recover from insults [54]–[56], we believe that the protection rendered by HSPB1 in these tissues could also be based on its capacity to maintain a functional MT network by facilitating MT formation.

In conclusion, our results unravel a novel function of HSPB1 on non-centrosomal MT formation, which brings new mechanistic insights into the ill-understood biology of mammalian non-centrosomal MT arrays and in the role of this chaperone in human diseases.

## Supporting Information

Figure S1 **Remaining acetylated MTs after nocodazole treatment.** (A) Representative images of HeLa HSPB1+ and HeLa HSPB1- cells 6 h after nocodazole treatment, immediately (without any repolymerization time) fixed and stained for α- and acetyl-tubulin. (B) Quantification of number and size of acetylated MTs from HSPB1+ and HSPB1- after nocodazole washout showed that there is no difference in remaining polymerization “seeds” between the different cell lines immediately upon depolymerization (n = 105 HeLa HSPB1+ cells and 110 HeLa HSPB1- cells). Data are presented as average ± SEM. Scale bar = 5 µm.
(TIF)

Figure S2 **CMT causing HSPB1 mutants induce the formation of de novo MTs to the same extent as wild-type HSPB1.** Representative image and quantification of *de novo* non-centrosomal MTs in HeLa cells expressing the S135F mutant. Cells were stained for α- and acetyl-tubulin. HeLa HSPB1 S135F cells presented a similar repolymerization phenotype as HeLa HSPB1+ cells (n = 41 cells for HeLa HSPB1 S135F). Data is presented as average ± SEM. Scale bar = 5 µm.
(TIF)

Figure S3 **MT repolymerization pattern of CHO cells.** Additional pictures from CHO HSPB1- and CHO HSPB1+ cells at 5 min after nocodazole washout. Note the large difference in aster size and number of non-centrosomal MTs between HSPB1− and HSPB1+ transfected CHO cells. Scale bar = 10 µm.
(TIF)

Figure S4 **Centrosomal asters are extremely stable in CHO cells after nocodazole treatment.** CHO cells after 6 h of nocodazole treatment (no recovery time) were stained for α- and γ-tubulin. Despite the extended nocodazole treatment, MT asters in these cells remained intact. Scale bar = 10 µm.
(TIF)

Figure S5 **HSPB1 levels and nocodazole treatment does not affect the expression and the architecture of the actin cytoskeleton.** (A) Western blot showing the expression of tubulin, actin and γ-tubulin in HeLa and CHO stable cell lines before and after nocodazole treatment. (B) Images of HeLa and CHO stables before nocodazole treatment stained for α-tubulin and F-actin (Phalloidin). (C) Nocodazole treatment depolymerizes MTs without affecting the F-actin network. We detected no differences in distribution or intensity of F-actin between cells lines or treatments. Scale bar = 10 µm.
(TIF)

Figure S6 **HSPB1 expression levels do not change the distribution of γ-tubulin in cells.** Images of HeLa and CHO stables 0 min after nocodazole treatment stained for α-tubulin and γ-tubulin. We detected no differences in distribution or intensity of these stainings between cells lines or treatments. Note that CHO cells have a more pronounced accumulation of γ-tubulin at the centrosome compared to HeLa cells. Scale bar = 10 µm.
(TIF)

Figure S7 **MT architecture at steady state in CHO cells expressing EB1-GFP.** Additional pictures of live cell imaging of CHO cells expressing EB1-GFP at time zero and maximum intensity projections of 20 frames (1 min). Note that CHO HSPB1- cells present a centrosomal polymerization pattern, as reflected by the highly concentrated and symmetrical, centrosomal-originating EB1-GFP tracks; while the pattern in CHO HSPB1+ cells is non-centrosomal, as reflected by the presence of dispersed EB1-GFP tracks originated from multiple non-centrosomal nucleation sites. These cells are also shown in Movie S3. Scale bar = 10 µm.
(TIF)

Figure S8 **GFP does not bind to polymerized MTs.** TEM micrograph from MTs polymerized in the presence of the negative control GFP. The density of MT-bound GFP was also quantified (0.17+/−0.27 GFPs/MT µm, n = 37 MTs, p<0.0001 in comparison with HSPB1). Scale bar = 200 nm.
(TIF)

Figure S9 **Integrated model for HSPB1 microtubule nucleation function and CMT pathogenesis.** During the formation of MTs, both WT and mutant HSPB1 bind to MTs enhancing their stability and their success rate of becoming a stable MT seed. When MTs reach a stable state, WT HSPB1 releases from the MTs and returns to its inactive, low affinity state. However, due to its increased binding capacity, the binding equilibrium of mutant HSPB1 has shifted and the protein remains bound to the MTs, leading to their enhanced stability.
(TIF)

Movie S1 **MT repolymerization in CHO cells.** CHO HSPB1− (top) and CHO HSPB1+ (bottom) were transfected with EB1-GFP, treated with nocodazole and washed while imaging with spinning disk confocal microscopy. Image z-stacks comprising the complete volume of the cells were acquired every 5 sec. The movie shows the maximum intensity projections of all slices in the z-stack with inverted grey scale. Images on the right are shown with overlayed EB1-GFP tracks. Note the large difference in the number of non-centrosomal and centrosomal MTs between cells.
(AVI)

Movie S2 **EB1-GFP tracks in steady state cells.** CHO HSPB1− (top) and CHO HSPB1+ (bottom) were transfected with EB1-GFP and imaged with spinning disk confocal microscopy. Image z-stacks comprising the centrosomal and Golgi region were acquired every 3 sec. The images on the left show the maximum intensity projections of all slices in the z-stack with inverted grey scale. Images on the right show these as cumulative maximum intensity projections over the elapsed time. Note the large number of non-centrosomal MTs formed in CHO-HSPB1+ cells.
(AVI)

Movie S3 **Additional movies showing EB1-GFP tracks in steady state cells.** Same experimental setup as Movie S2. The two top rows show HSPB1− cells while the two bottom rows show HSPB1+ cells.
(AVI)

Table S1 HSPB1 levels do not interfere with MT dynamics in steady state cells. TUBB3-GFP MTs were imaged at 3 s intervals in naive HeLa cells or in cells overexpressing HSPB1 or with HSPB1 knockdown transiently transfected with TUBB3-GFP. The MTs life histories are represented as the percentage of time spent in growth, shrinkage or pause phase. Catastrophe frequency is given as the frequency that MTs transitioned from growth (or pause) to shrinkage. Rescue frequency corresponds to the frequency at which MTs transitioned from shrinkage (or pause) to growth. Data are presented as average ± SD. No significant differences between the different cell lines were found.
(DOC)
