# Supplementary figures and images for: HSPB1 Facilitates the Formation of Non-Centrosomal Microtubules
Source: PLoS One. 2013 Jun 24;8(6):e66541. doi: 10.1371/journal.pone.0066541 (PMC3691211; doi:10.1371/journal.pone.0066541)

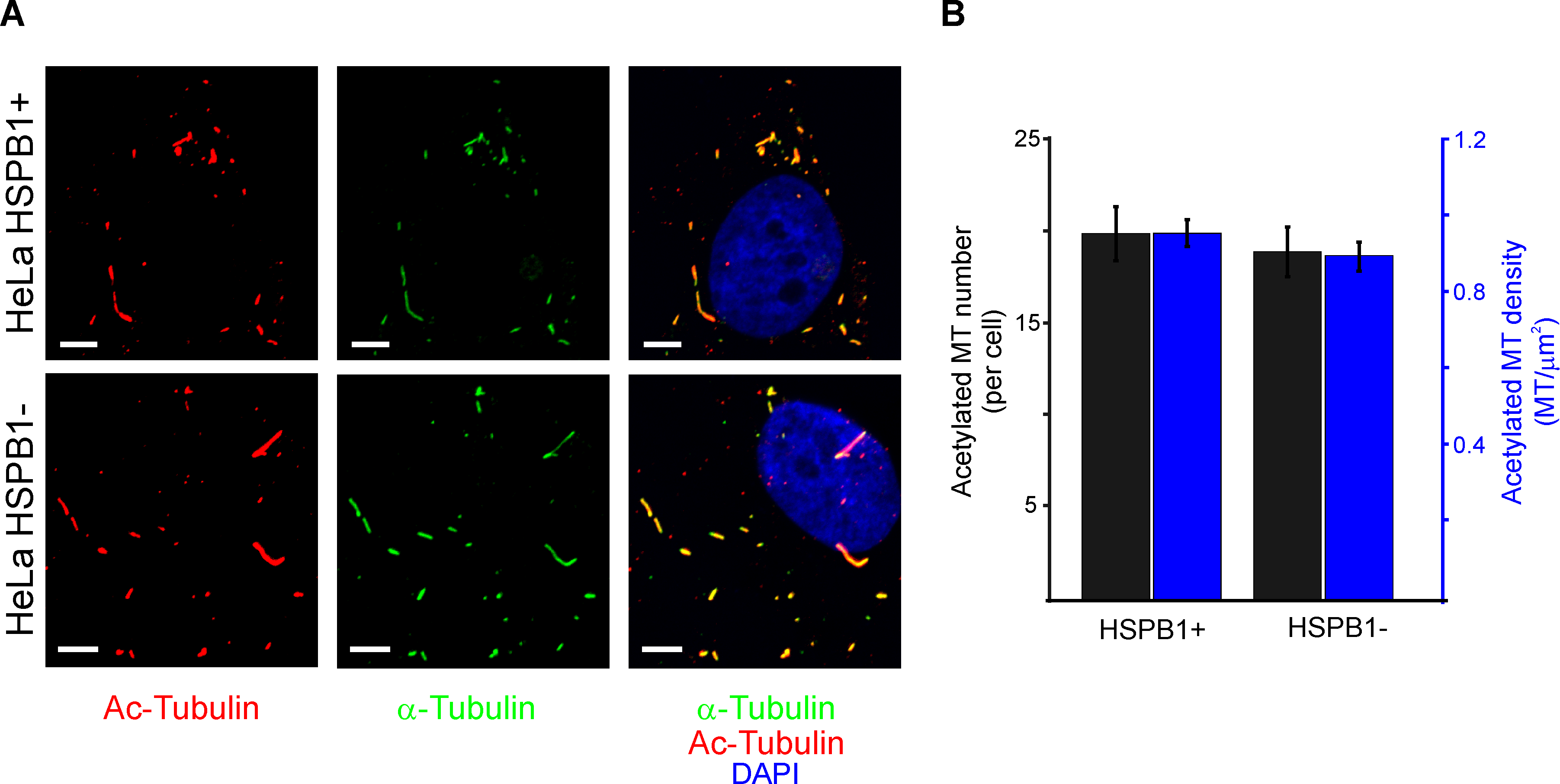

Supplement: Figure S1 — Remaining acetylated MTs after nocodazole treatment. (A) Representative images of HeLa HSPB1+ and HeLa HSPB1- cells 6 h after nocodazole treatment, immediately (without any repolymerization time) fixed and stained for α- and acetyl-tubulin. (B) Quantification of number and size of acetylated MTs from HSPB1+ and HSPB1- after nocodazole washout showed that there is no difference in remaining polymerization “seeds” between the different cell lines immediately upon depolymerization (n = 105 HeLa HSPB1+ cells and 110 HeLa HSPB1- cells). Data are presented as average ± SEM. Scale bar = 5 µm. (TIF) [file pone.0066541.s001.tif]

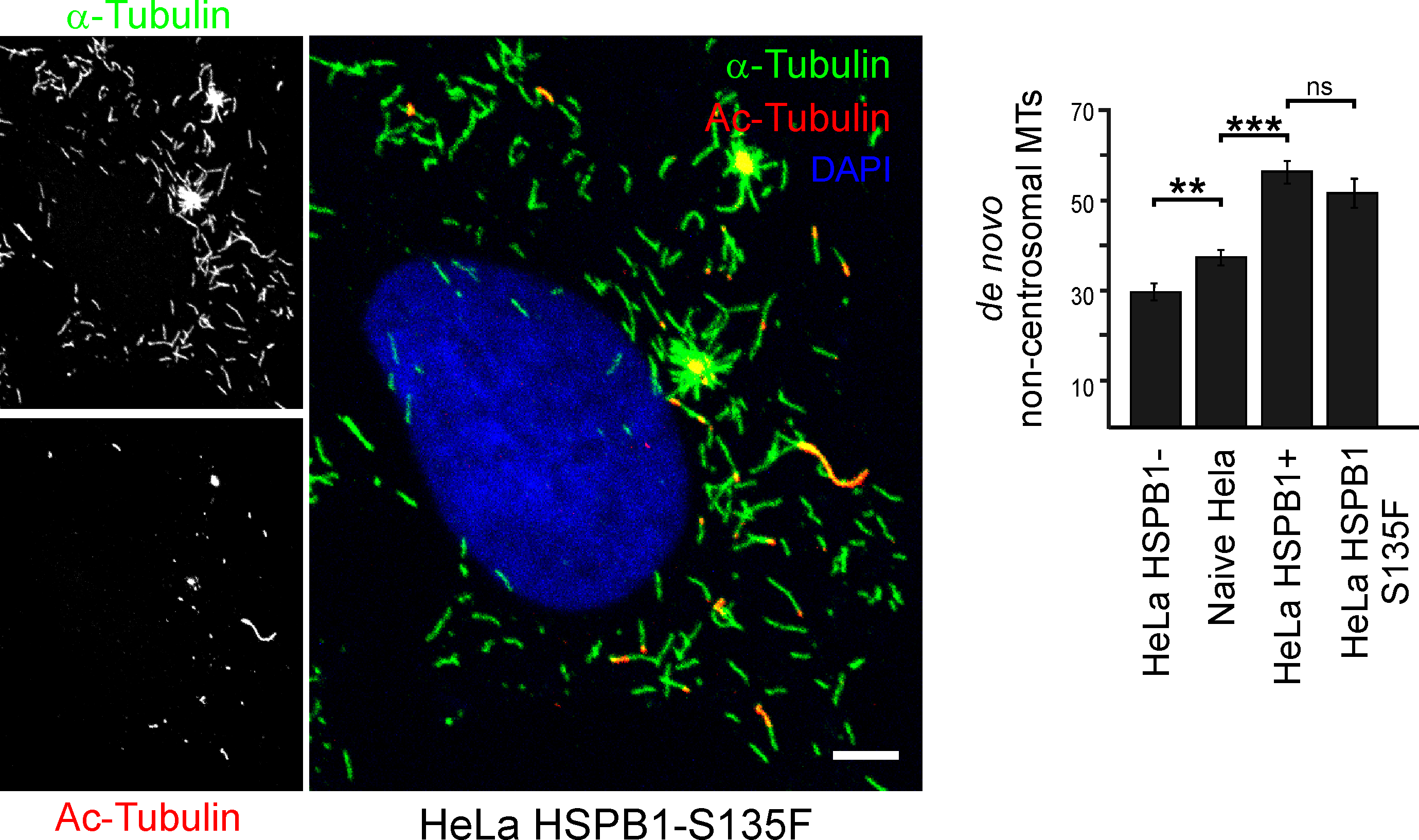

Supplement: Figure S2 — CMT causing HSPB1 mutants induce the formation of de novo MTs to the same extent as wild-type HSPB1. Representative image and quantification of de novo non-centrosomal MTs in HeLa cells expressing the S135F mutant. Cells were stained for α- and acetyl-tubulin. HeLa HSPB1 S135F cells presented a similar repolymerization phenotype as HeLa HSPB1+ cells (n = 41 cells for HeLa HSPB1 S135F). Data is presented as average ± SEM. Scale bar = 5 µm. (TIF) [file pone.0066541.s002.tif]

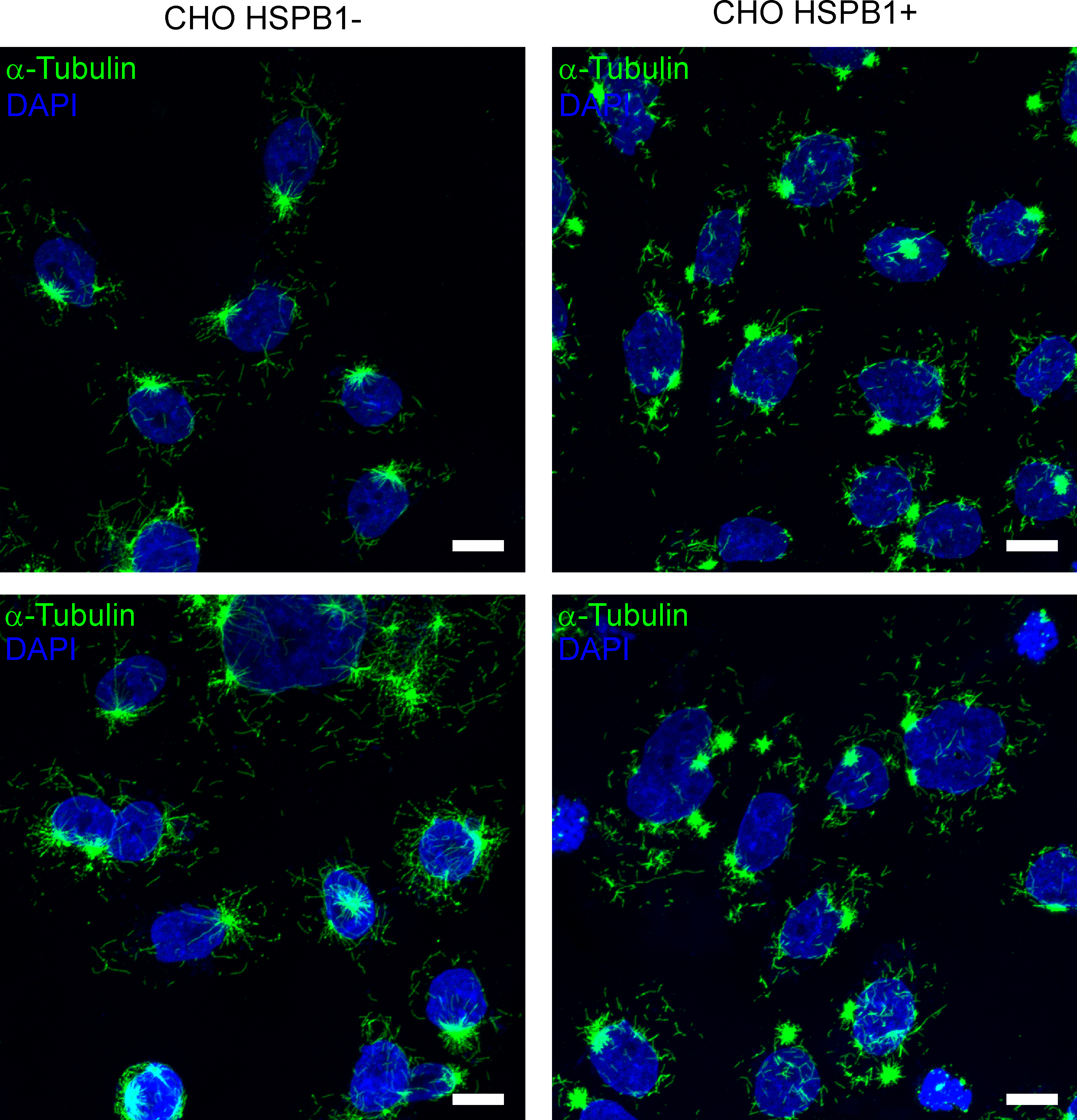

Supplement: Figure S3 — MT repolymerization pattern of CHO cells. Additional pictures from CHO HSPB1- and CHO HSPB1+ cells at 5 min after nocodazole washout. Note the large difference in aster size and number of non-centrosomal MTs between HSPB1− and HSPB1+ transfected CHO cells. Scale bar = 10 µm. (TIF) [file pone.0066541.s003.tif]

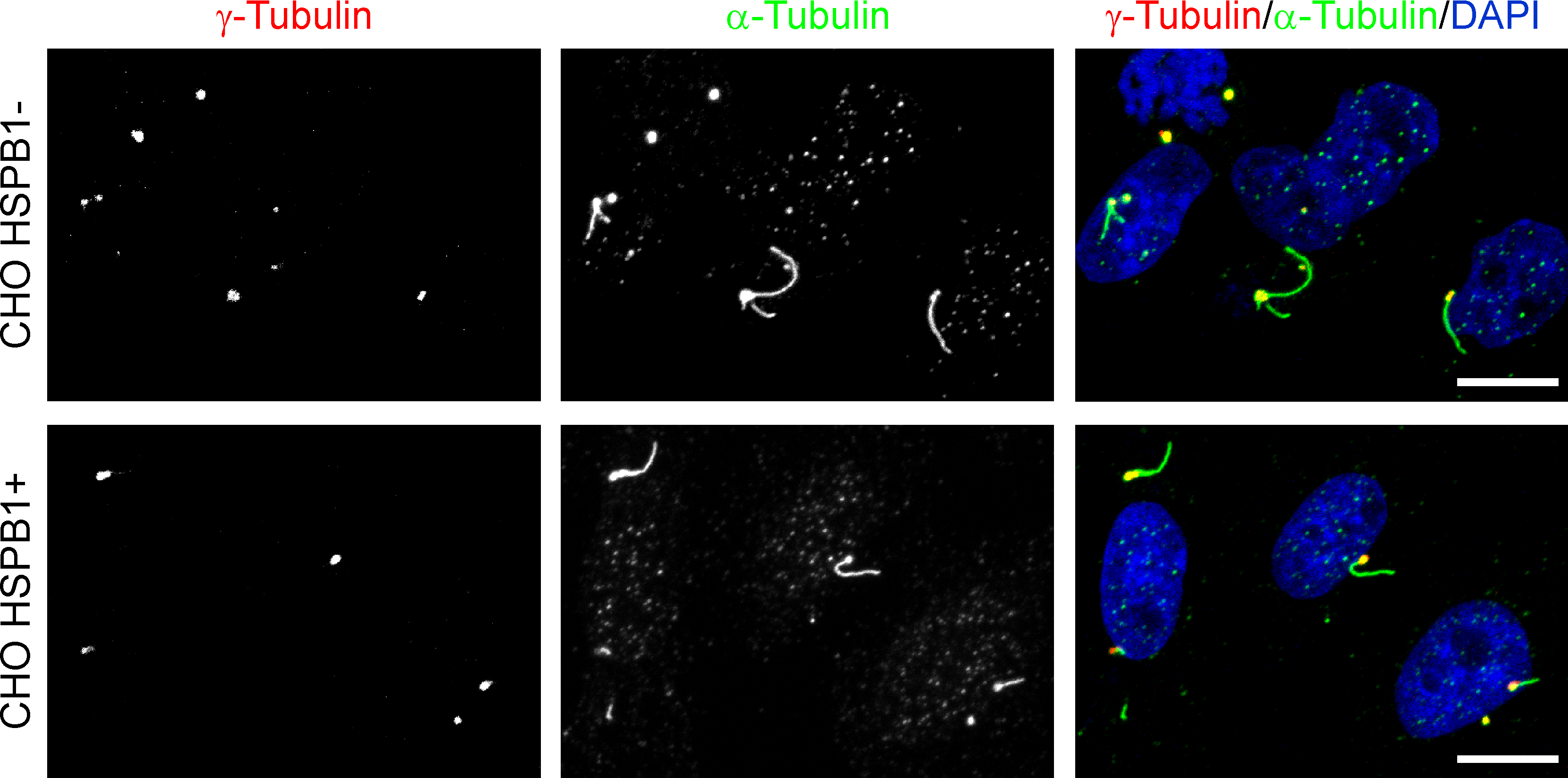

Supplement: Figure S4 — Centrosomal asters are extremely stable in CHO cells after nocodazole treatment. CHO cells after 6 h of nocodazole treatment (no recovery time) were stained for α- and γ-tubulin. Despite the extended nocodazole treatment, MT asters in these cells remained intact. Scale bar = 10 µm. (TIF) [file pone.0066541.s004.tif]

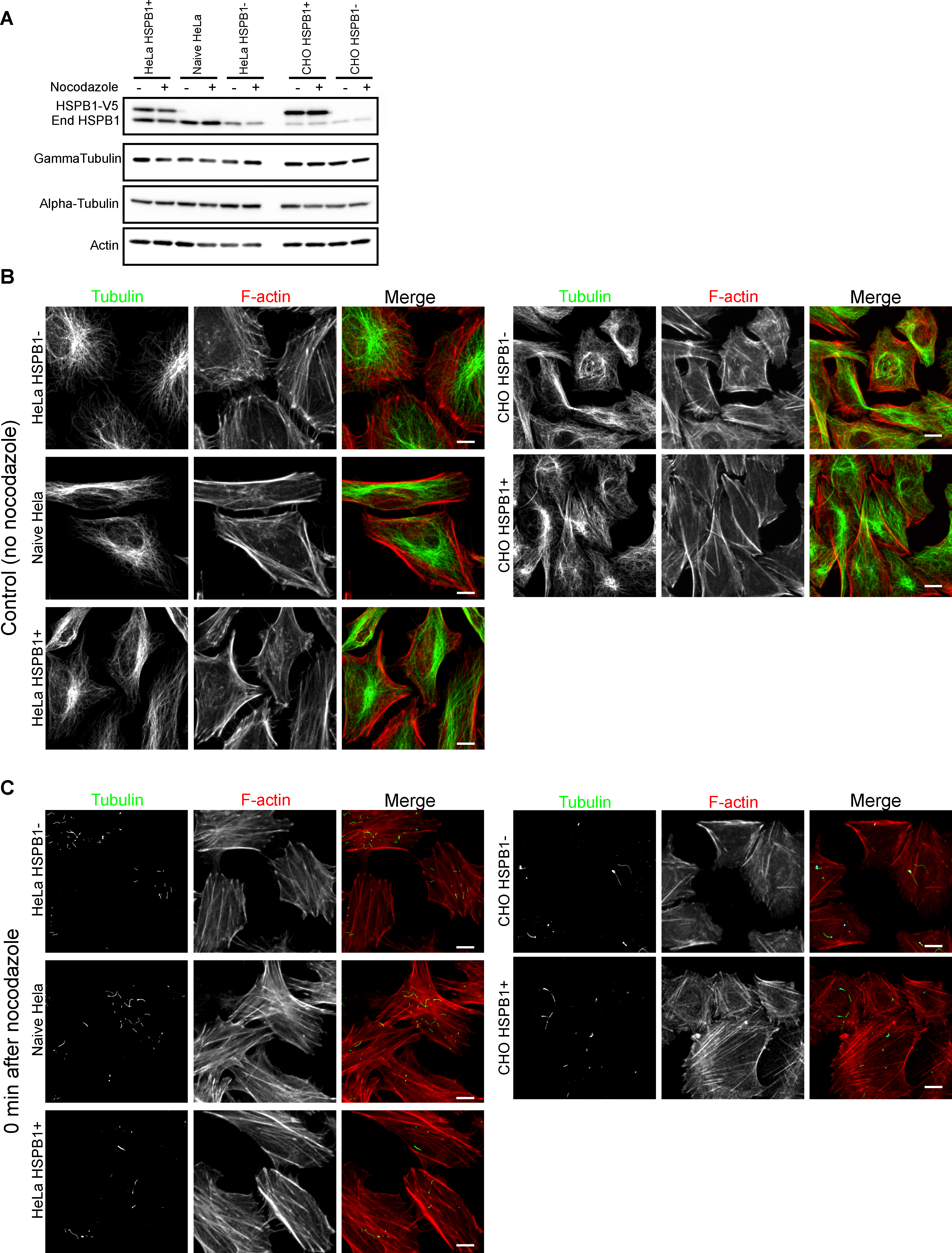

Supplement: Figure S5 — HSPB1 levels and nocodazole treatment does not affect the expression and the architecture of the actin cytoskeleton. (A) Western blot showing the expression of tubulin, actin and γ-tubulin in HeLa and CHO stable cell lines before and after nocodazole treatment. (B) Images of HeLa and CHO stables before nocodazole treatment stained for α-tubulin and F-actin (Phalloidin). (C) Nocodazole treatment depolymerizes MTs without affecting the F-actin network. We detected no differences in distribution or intensity of F-actin between cells lines or treatments. Scale bar = 10 µm. (TIF) [file pone.0066541.s005.tif]

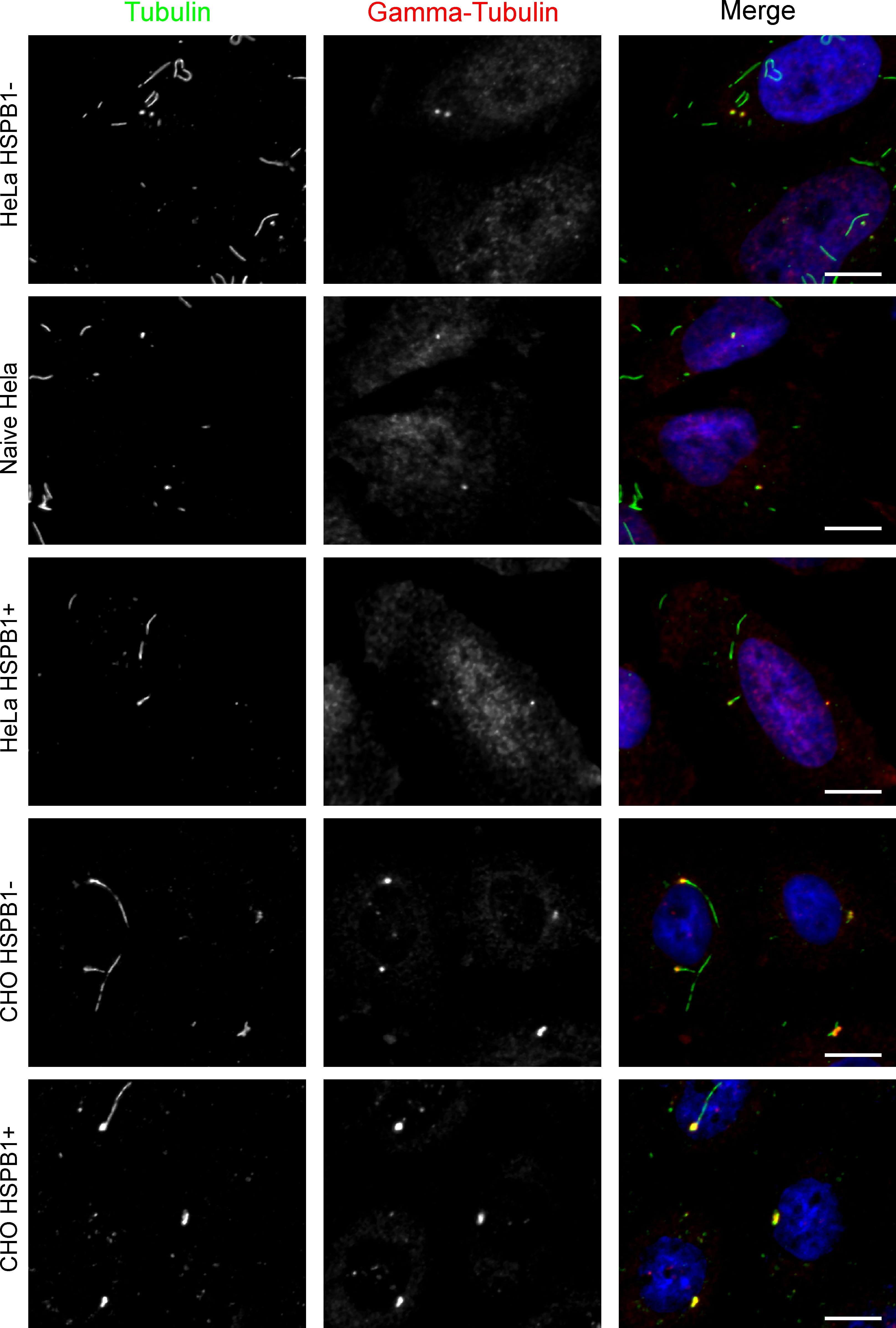

Supplement: Figure S6 — HSPB1 expression levels do not change the distribution of γ-tubulin in cells. Images of HeLa and CHO stables 0 min after nocodazole treatment stained for α-tubulin and γ-tubulin. We detected no differences in distribution or intensity of these stainings between cells lines or treatments. Note that CHO cells have a more pronounced accumulation of γ-tubulin at the centrosome compared to HeLa cells. Scale bar = 10 µm. (TIF) [file pone.0066541.s006.tif]

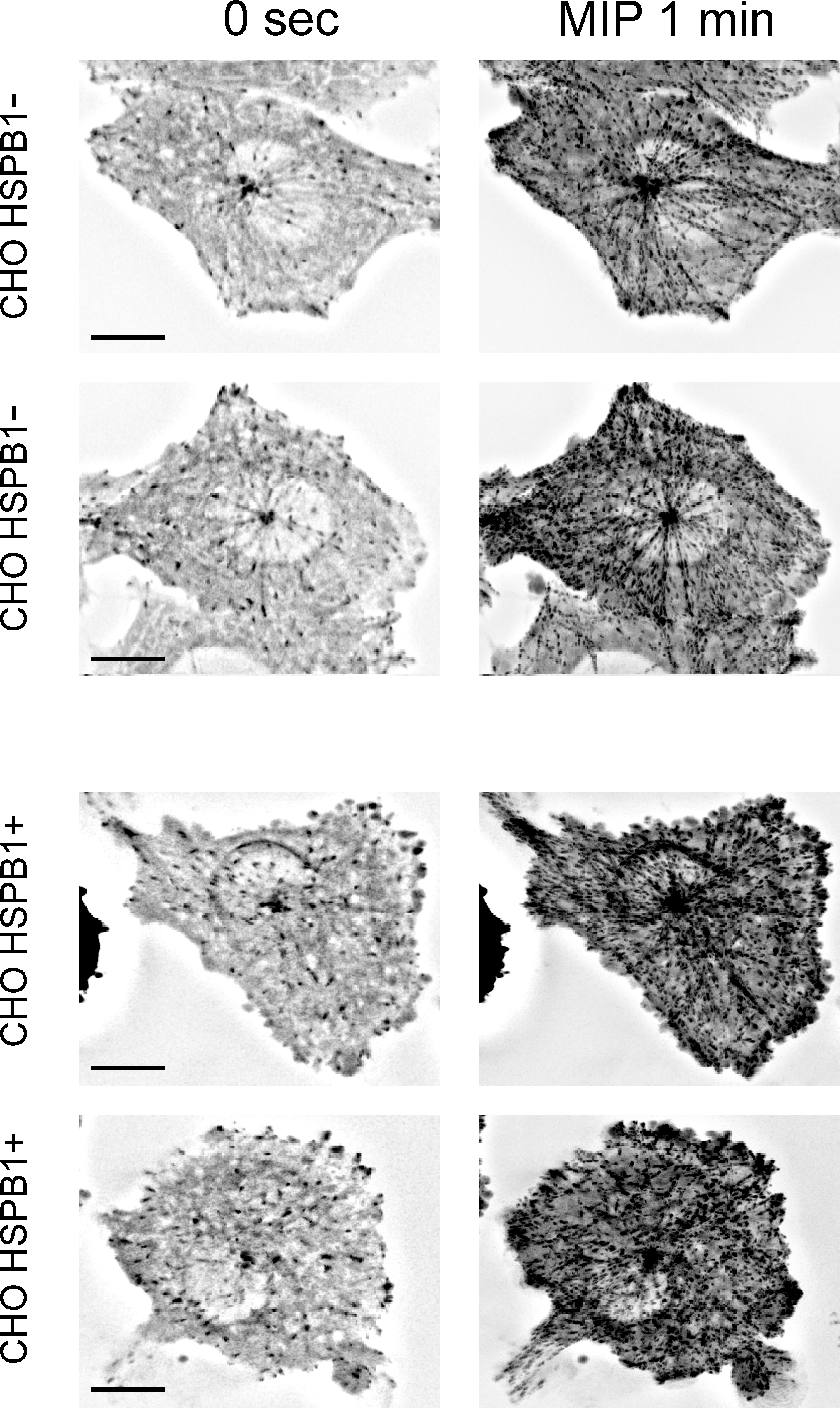

Supplement: Figure S7 — MT architecture at steady state in CHO cells expressing EB1-GFP. Additional pictures of live cell imaging of CHO cells expressing EB1-GFP at time zero and maximum intensity projections of 20 frames (1 min). Note that CHO HSPB1- cells present a centrosomal polymerization pattern, as reflected by the highly concentrated and symmetrical, centrosomal-originating EB1-GFP tracks; while the pattern in CHO HSPB1+ cells is non-centrosomal, as reflected by the presence of dispersed EB1-GFP tracks originated from multiple non-centrosomal nucleation sites. These cells are also shown in Movie S3. Scale bar = 10 µm. (TIF) [file pone.0066541.s007.tif]

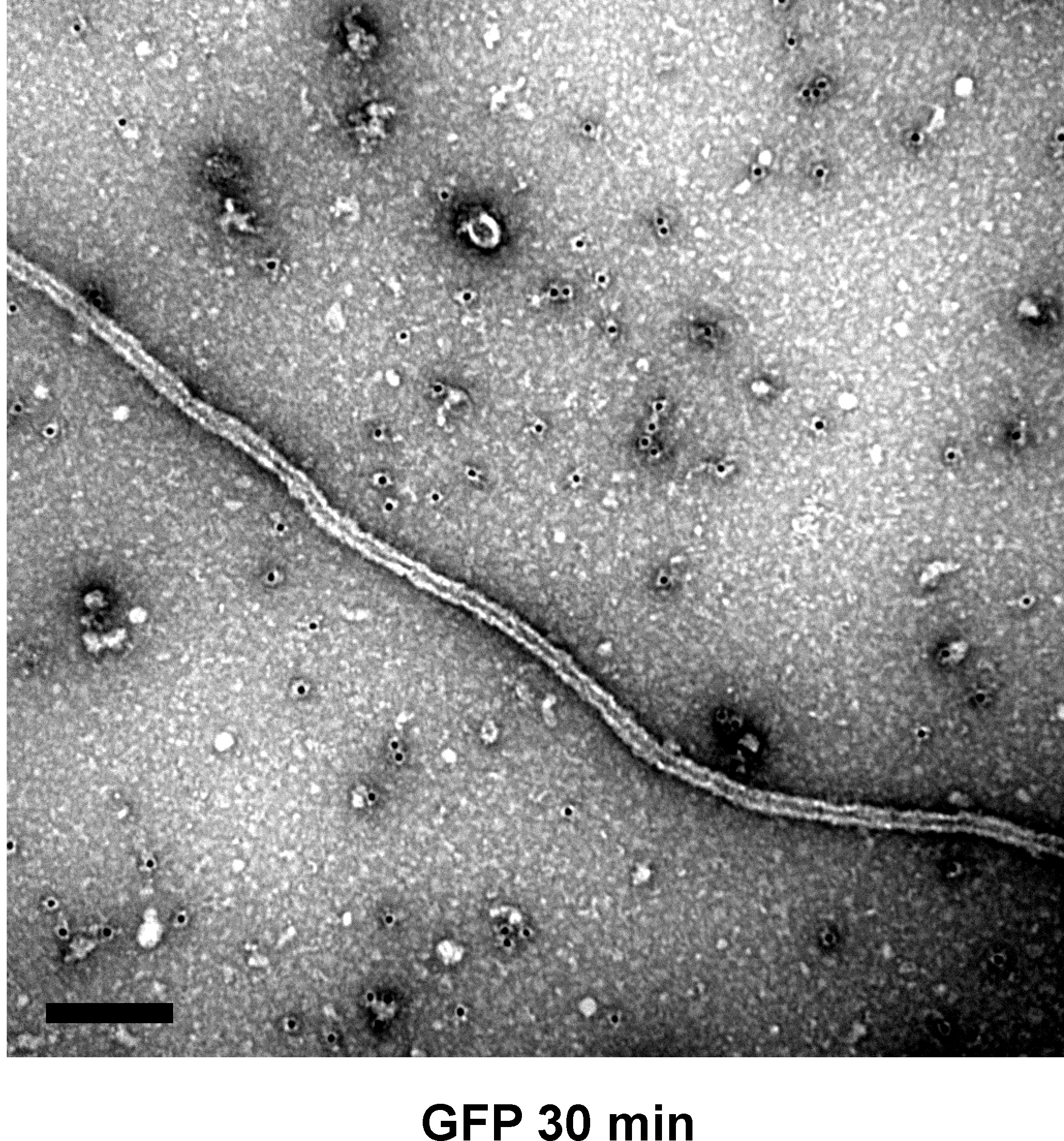

Supplement: Figure S8 — GFP does not bind to polymerized MTs. TEM micrograph from MTs polymerized in the presence of the negative control GFP. The density of MT-bound GFP was also quantified (0.17+/−0.27 GFPs/MT µm, n = 37 MTs, p<0.0001 in comparison with HSPB1). Scale bar = 200 nm. (TIF) [file pone.0066541.s008.tif]

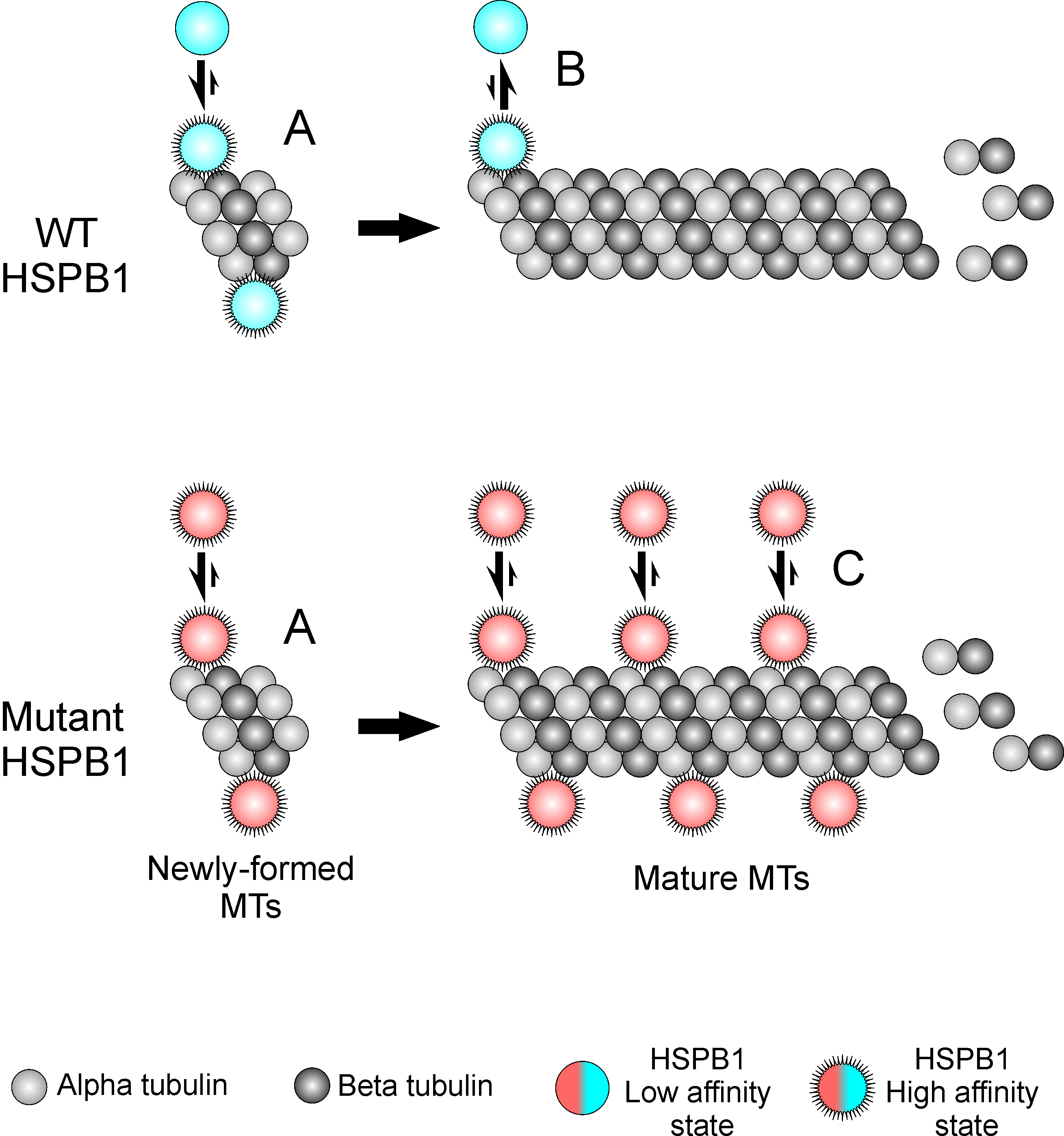

Supplement: Figure S9 — Integrated model for HSPB1 microtubule nucleation function and CMT pathogenesis. During the formation of MTs, both WT and mutant HSPB1 bind to MTs enhancing their stability and their success rate of becoming a stable MT seed. When MTs reach a stable state, WT HSPB1 releases from the MTs and returns to its inactive, low affinity state. However, due to its increased binding capacity, the binding equilibrium of mutant HSPB1 has shifted and the protein remains bound to the MTs, leading to their enhanced stability. (TIF) [file pone.0066541.s009.tif]
