# Supplementary material for: HSPB1 Facilitates the Formation of Non-Centrosomal Microtubules
Source: PLoS One. 2013 Jun 24;8(6):e66541. doi: 10.1371/journal.pone.0066541 (PMC3691211; doi:10.1371/journal.pone.0066541)
Supplement: Table S1 — HSPB1 levels do not interfere with MT dynamics in steady state cells. TUBB3-GFP MTs were imaged at 3 s intervals in naive HeLa cells or in cells overexpressing HSPB1 or with HSPB1 knockdown transiently transfected with TUBB3-GFP. The MTs life histories are represented as the percentage of time spent in growth, shrinkage or pause phase. Catastrophe frequency is given as the frequency that MTs transitioned from growth (or pause) to shrinkage. Rescue frequency corresponds to the frequency at which MTs transitioned from shrinkage (or pause) to growth. Data are presented as average ± SD. No significant differences between the different cell lines were found. (DOC) [file pone.0066541.s013.doc]

Table S1 - HSPB1 levels do not interfere with MT dynamics in steady state cells.

|  | **HeLa HSPB1 -**  (n= 48 MTs) | **Naive HeLa**  (n= 42 MTs) | **HeLa HSPB1 +**  (n= 47 MTs) |
| --- | --- | --- | --- |
| **% Growth** | 11.5 (± 10.0) | 11.1 (± 6.1) | 14.3 (± 9.5) |
| **% Shrinkage** | 14.5 (± 9.6) | 13.8 (± 8.7) | 14.7 (± 12.2) |
| **% Pause** | 74.1 (± 15.7) | 75.1 (± 11.9) | 71.1 (± 15.3) |
| **Growth velocity (µm/s)** | 0.279 (± 0.149) | 0.221 (± 0.081) | 0.227 (± 0.082) |
| **Shrink velocity (µm/s)** | 0.260 (± 0.110) | 0.253 (± 0.115) | 0.261 (± 0.104) |
| **Catastrophe freq. (s^-1^)** | 0.160 (± 0.078) | 0.172 (± 0.091) | 0.153 (± 0.096) |
| **Rescue freq. (s^-1^)** | 0.138 (± 0.090) | 0.090 (± 0.064) | 0.180 (± 0.091) |

TUBB3-GFP MTs were imaged at 3s intervals in naive HeLa cells or in cells overexpressing HSPB1 or with HSPB1 knockdown transiently transfected with TUBB3-GFP. The MTs life histories are represented as the percentage of time spent in growth, shrinkage or pause phase. Catastrophe frequency is given as the frequency that MTs transitioned from growth (or pause) to shrinkage. Rescue frequency corresponds to the frequency at which MTs transitioned from shrinkage (or pause) to growth. Data are presented as average ± SD. No significant differences between the different cell lines were found.
